# Impact of demography and population dynamics on the genetic architecture of human longevity

**DOI:** 10.18632/aging.101515

**Published:** 2018-08-08

**Authors:** Cristina Giuliani, Marco Sazzini, Chiara Pirazzini, Maria Giulia Bacalini, Elena Marasco, Guido Alberto Gnecchi Ruscone, Fang Fang, Stefania Sarno, Davide Gentilini, Anna Maria Di Blasio, Paolina Crocco, Giuseppe Passarino, Daniela Mari, Daniela Monti, Benedetta Nacmias, Sandro Sorbi, Carlo Salvarani, Mariagrazia Catanoso, Davide Pettener, Donata Luiselli, Svetlana Ukraintseva, Anatoliy Yashin, Claudio Franceschi, Paolo Garagnani

**Affiliations:** 1 Department of Biological, Geological, and Environmental Sciences (BiGeA), Laboratory of Molecular Anthropology and Centre for Genome Biology, University of Bologna, Bologna, Italy; 2 School of Anthropology and Museum Ethnography, University of Oxford, Oxford, UK; 3 Interdepartmental Center "L. Galvani," (CIG), University of Bologna, Bologna, Italy; 4 IRCCS, Institute of Neurological Sciences of Bologna, Bologna, Italy; 5 Department of Experimental, Diagnostic and Specialty Medicine (DIMES), University of Bologna, Bologna, Italy; 6 Applied Biomedical Research Center (CRBA), S. Orsola-Malpighi Polyclinic, Bologna, Italy; 7 Biodemography of Aging Research Unit, Social Science Research Institute, Duke University, Durham, NC 27708, USA; 8 Istituto Auxologico Italiano IRCCS, Cusano Milanino, Milan, Italy; 9 Department of Biology, Ecology and Earth Sciences, University of Calabria, Rende, Italy; 10 Geriatric Unit, Department of Medical Sciences and Community Health, University of Milan, Milan, Italy; 11 Fondazione Ca' Granda, IRCCS Ospedale Maggiore Policlinico, Milan, Italy; 12 Department of Experimental and Clinical Biomedical Sciences "Mario Serio", University of Florence, Florence, Italy; 13 Department of Neuroscience, Psychology, Drug Research and Child Health, University of Florence, Florence, Italy; 14 IRCCS Don Gnocchi, Florence, Italy; 15 Azienda Ospedaliera-IRCCS, Reggio Emilia, Italy; 16 Department of Surgical, Medical, Dental and Morphological Sciences with Interest transplant, Oncological and Regenerative Medicine, Università di Modena e Reggio Emilia, Italy; 17 Department for the Cultural Heritage (DBC), University of Bologna, Ravenna, Italy; 18 Clinical Chemistry, Department of Laboratory Medicine, Karolinska Institutet at Huddinge University Hospital, S-141 86 Stockholm, Sweden; 19 CNR Institute of Molecular Genetics, Unit of Bologna, Bologna, Italy; 20 Rizzoli Orthopaedic Institute, Laboratory of Cell Biology, Bologna, Italy; 21 Co-senior authors; * Equal contribution

**Keywords:** centenarians, population dynamics, longevity, human genetics

## Abstract

The study of the genetics of longevity has been mainly addressed by GWASs that considered subjects from different populations to reach higher statistical power. The "price to pay" is that population-specific evolutionary histories and trade-offs were neglected in the investigation of gene-environment interactions. We propose a new “diachronic” approach that considers processes occurred at both evolutionary and lifespan timescales. We focused on a well-characterized population in terms of evolutionary history (*i.e.* Italians) and we generated genome-wide data for 333 centenarians from the peninsula and 773 geographically-matched healthy individuals. Obtained results showed that: (i) centenarian genomes are enriched for an ancestral component likely shaped by pre-Neolithic migrations; (ii) centenarians born in Northern Italy unexpectedly clustered with controls from Central/Southern Italy suggesting that Neolithic and Bronze Age gene flow did not favor longevity in this population; (iii) local past adaptive events in response to pathogens and targeting arachidonic acid metabolism became favorable for longevity; (iv) lifelong changes in the frequency of several alleles revealed pleiotropy and trade-off mechanisms crucial for longevity. Therefore, we propose that demographic history and ancient/recent population dynamics need to be properly considered to identify genes involved in longevity, which can differ in different temporal/spatial settings.

## Introduction

The new industrial/socio-economic revolution that started in the last century led to a dramatic change in the relationship between humans and their environment, and population ageing represents one of its main byproducts. Longevity itself could be viewed as a product of complex biological, social and cultural interconnections, in which internal and external environments (both individual- and population-specific) may have a dominant impact on the final outcome [1,2].

Longevity is also related to complex diseases with late age at onset [3–6], though this relationship may sometimes involve antagonistic pleiotropy or trade-offs [7,8]. Interestingly, tradeoffs occur when a trait cannot increase without a decrease in another one and it has been supposed that beneficial genotypes may pleiotropically generate deleterious effects in phenotypes that trade off with them. This concept has been proposed for many diseases, such as cancer, neurodegeneration, autoimmune and infectious diseases [9]. It has been also suggested that an evolutionary approach that relies on models/assumptions of population genetics may help in predicting the genetic background of susceptibility to such diseases [6,10–12]. Following this rationale, we have recently investigated the evolutionary history of the Italian population [13] to obtain prior knowledge essential to carry out the present study. In particular, we showed that genetic variants conferring increased susceptibility to certain diseases have been maintained in the Italian gene pool due to their past adaptive role and that their frequency varies along a latitudinal cline also in relation to the peculiar demographic histories of the examined Italian groups [13].

These findings corroborated the emerging belief that micro-evolutionary mechanisms influencing genetic diversity at the population level should not be neglected in studies focused on human disease and longevity [14]. In fact, it is well-known that population dynamics (e.g. migration, demographic expansion/bottlenecks, admixture and adaptive events) that occurred in a timeframe of thousands of years could influence the distribution of both risk and protective variants [15]. Moreover, these processes may also lead to substantial differences in linkage disequilibrium (LD) patterns that could explain non-replication of genetic associations found in traditional genome-wide association studies (GWAS) across different cohorts [16,17]. In fact, many recent GWAS and meta-analyses included multiple populations, which might share only a limited fraction of their genetic ancestry, and thus need to be interpreted with caution [18]. Allele frequencies are indeed the result of a complex interplay between gene-environment interaction and demographic processes [19], which may differ even among apparently closely-related human groups.

By considering all these issues, longevity could be viewed as a product of integration of the effects of evolutionary processes with those that occurred during the individual’s lifespan, as proposed by the “mutation accumulation” [20] and the “antagonistic pleiotropy” evolutionary hypotheses [21]. Nevertheless, only one recent paper has attempted to test these theories using genome-wide data [22], and a substantial research effort is still needed to effectively implement this theoretical approach into the study of human longevity [23].

We therefore adopted this innovative conceptual framework to investigate the genetic factors that contributed to the evolution of longevity in the Italian population. We relied on the assumption that this complex phenotype may result from the interplay between the evolutionary history specific of a given population and the genetic trade-offs to which single individuals are subjected during different phases of their life, and which could also vary according to birth cohorts [24]. Overall, the following demographic, biological and environmental dynamics were considered to jointly drive the evolution of genetic backgrounds compatible with human longevity:

1**)** past population processes (e.g. migrations, admixture and/or adaptive events) that are peculiar of each human group and have shaped the centenarians’ genetic profiles, as they are part of their population of origin;

2) changing internal and external environments that have then differentially interacted with such a substratum over the individuals’ lifespan and according to the different phases of life, which are characterized by substantial modifications in diet, disease susceptibility, stress stimuli, hormone profile, and metabolism. Due to the industrial revolution and the onward globalization processes, this issue has gained particular relevance in the very recent history of western societies, for which the Italian population is proposed to be highly representative.

A diachronic approach able to combine information about processes occurred at these different timescales (*i.e.* evolutionary and lifespan ones) is thus expected to improve the understanding of the genetic bases of human longevity. We therefore applied it to the study of a highly selected cohort of people more than 100 years old and belonging to a population (*i.e*. the Italian one) whose evolutionary history has been extensively investigated.

The aim of this study is consequently twofold: (i) to test whether past migrations, admixture and/or local adaptations may have influenced the distribution of variants involved in human longevity in the Italian population; (ii) to explore patterns of genomic variation in groups of individuals with different ages and, especially, in centenarians, to pinpoint possible pleiotropic effects and changing gene-environment interactions of longevity-related loci.

For this purpose, we generated genome-wide data for 333 centenarians sampled from Northern, Central and Southern Italy. We then compared them with data available for 773 healthy individuals (age range 19-85 yrs) who have been demonstrated to be highly representative of the overall Italian population [13,25].

## RESULTS AND DISCUSSION

### Evolutionary dynamics and population history

**Figure 1:**
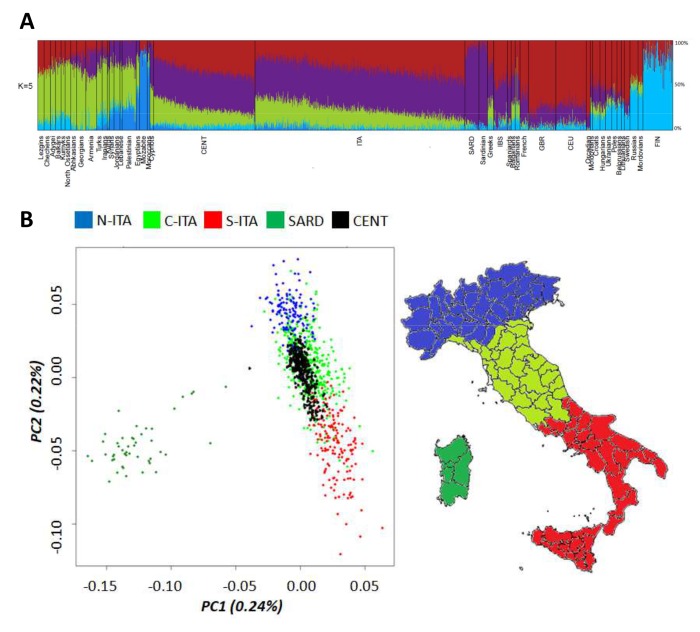
(**A**) Ancestry proportions at K = 5 estimated by ADMIXTURE analysis performed on Italian centenarians and controls, as well as on 50 Mediterranean and European populations. (**B**) First and second PCs calculated on the Italian general population (controls). Centenarians were projected as a supplementary group. Individuals from Northern Italy are indicated in blue, from Central Italy in green and from Southern Italy in red. Individuals from Sardinia are displayed in dark green.

The entire Italian dataset (*i.e.* centenarians and controls) was first submitted to ADMIXTURE analysis (Figure 1A) by considering also data for 50 Mediterranean/European populations retrieved from public databases. As expected, centenarians’ ancestry fractions fitted within the range of variation observed for the Italian and other considered populations. The only exception was accounted by the genetic component more represented in Northeastern Europe (light blue), which is slightly increased in centenarians with respect to Italian controls (3.8% vs. 2.3%, p-value = 2*10^-5^). Previous studies suggested that such a component represents the relic of an ancient genomic background that might be less spread in Southern Europe with respect to northern regions just during the Paleolithic [26]. The contribution of this ancestry fraction to the southern European gene pool was then further reduced by the introduction of additional genetic components throughout migrations from the Middle East after the Last Glacial Maximum (LGM) and during the Epigravettian transition [27,28]. Distribution of this genetic component in the overall Italian population could have been also shaped by post LGM re-expansion of human groups along the peninsula from glacial refugia presumably located in Central/Southern Italy [29]. Interestingly, it is also similarly represented in centenarians and Sardinians (3.8% vs. 3.9%, p-value *ns*), which are supposed to have escaped Late Neolithic and post-Neolithic genetic reshuffling [30], thus corroborating the hypothesis of a considerably ancient ancestry fraction. Nevertheless, no genes involved in longevity were found to be enriched when considering the 1,894 SNPs informative of this genetic signature.

To deepen the resolution of our analysis, we restricted the comparison between centenarians and the overall Italian population to a micro-geographical scale. Principal Component Analysis (PCA) showed that centenarians fitted into the previously described patterns of clear differentiation between peninsular Italians and Sardinians described by PC1, as well as of the latitudinal cline of variation pointed out by PC2 [13,31]. However, they seemed to be more homogeneous (*i.e.* less scattered along PC2) than controls and turned out to overlap mainly with individuals from Central and Southern Italy (Figure 1B), despite their self-reported origins encompassed both Northern, Central and Southern Italian ones. This is suggestive of a peculiar genetic signature mainly represented in Central/Southern Italians and particularly enriched in centenarians, but not directly ascribable to a single ancestry component among those identified by ADMIXTURE analysis.

The observed patterns of differentiation between centenarians and controls were confirmed via a Discriminant Analysis of Principal Components (DAPC) by computing posterior membership probabilities for each individual to belong to the previously described Italian population clusters [13]. Posterior membership probabilities for each centenarian were reported in relation to their place of birth. Concerning centenarians recruited in Northern Italy, only 73% of them was actually born in this geographical area, as expected by migration patterns typical of the recent Italian demographic history. This might represent a first issue partially responsible for the observed close genetic relationship between centenarians and Central/Southern Italians. Nevertheless, also 84% and 10% of the centenarians born in Northern Italy showed appreciable genetic similarity to people respectively from Central and Southern Italy, with only 6% of them being assigned to the Northern Italian cluster. Most of the centenarians recruited in Central Italy were born in such a macro-geographical area (96%), with 42% of them being confirmed to be genetically close to Central Italians and 58% being instead assigned to the Southern Italian group according to the calculated membership probabilities. Finally, all centenarians recruited in Southern Italy are born in the southern regions of the peninsula, but 51% of them were assigned by DAPC to the Central Italian group and 49% to the Southern Italian one.

A possible explanation for such a peculiar pattern is that recent ancestors of most centenarians born in Northern Italy have previously migrated from central/southern regions. Unfortunately, we have no genealogical data for the recruited individuals to formally test this hypothesis. A second explanation is instead that a set of genetic variants sufficiently ancient to be distributed across the entire Italian population, but present at higher frequency in central/southern groups with respect to northern ones, could contribute to increased probability to develop the longevity phenotype and is thus enriched in centenarians irrespectively of their recent micro-geographical origin. Although the proposed hypotheses could not be mutually exclusive, the latter assumption seems to fit better with a maximum parsimony criterion by guessing that an *ancestral* fraction of the Italian genetic background may act as one of the favourable prerequisites to become centenarian. The peculiar distribution of such a genetic component in the Italian population may stem from its ancient origin and suggests its potential correlation with a series of post-LGM population movements associated to the re-expansion of human groups along the Italian peninsula from central/southern glacial refugia [29]. Subsequent Neolithic migrations that reached Italy [32,33] and, especially, the massive Late Neolithic and Bronze Age demographic processes that reshuffled the genomic landscape mainly of Northern Italians, then plausibly diluted these pre-Neolithic contributions. This had potentially laid the foundation for present-day underrepresentation of such an ancestral component in Northern Italy.

Moreover, population movements from Continental Europe are also supposed to have prompted local adaptations of Northern Italian groups to temperate climate conditions, emphasizing their divergent adaptive trajectory with respect to Central and Southern Italians, which instead received gene flow mainly from Mediterranean populations and were found to be more subjected to pathogen-related selective pressures [13]. To test whether, in addition to the described peculiar demographic history, some of these adaptive events may have shaped traits useful also to reach extreme longevity, we evaluated the association of this phenotype to loci targeted by positive selection in the population groups distributed along the Italian peninsula. For this purpose, we performed a GWAS by comparing centenarians and Italian controls and we searched for the recently identified top candidate adaptive SNPs [13] (46 SNPs, Table 1S) among variants that resulted significantly associated to longevity-.

The quantile-quantile (QQ) plot of association results demonstrated no genomic inflation (λ = 1.02), as showed in Figure 1S-A. As expected according to the complexity of the investigated phenotype, no strong associations were observed at the genome-wide level (Figure 1S-B). However, promising small-effect loci showing nominal significance were identified (p-value < 1*10^-4^) and reported in Table 2S, including genes already pointed out by the Health and Retirement Study (HRS) (Supplementary description - Part I). Since our cohort of centenarians was made up by 258 females and 75 males, the obtained results revealed genes and SNPs likely involved in female longevity. In fact, we performed the same analysis only on females and it confirmed results obtained by considering all centenarians and the sex as a covariate, while we did not replicate the analysis only on males because of their small sample size (N = 75). Among SNPs significantly associated to longevity (p-value < 1*10^-4^), six (rs2111720, rs1127102, rs3739704, rs1053959, rs3116602, rs3118914) showed also relevant signatures of positive selection in the Italian population. The most significant one was rs1053959 (exm772339, iHS: S_ITA = 3.67, C_ITA = 3.25, N_ITA = 2.91; p-value_cent-ctrl_ = 5.97*10^-3^), which is located on the *PTGR1* gene. This variant represents an eQTL and is known to affect PTGR1 expression in different tissues (Table 3S), with the CC genotype being associated to increased gene expression, the CA one to intermediate values and the AA genotype to reduced levels of *PTGR1* expression. The C allele, which has been targeted by positive selection in Italians plausibly according to its capability to restrict inflammation and to confer increased resistance to *Mycobacterium tuberculosis* [13] is more represented in centenarians than in controls. The PTGR1 protein is a prostaglandin reductase 1 and catalyzes the conversion of leukotriene B4, a well-known pro-inflammatory mediator, into its biological less active metabolite. Leukotriene B4 is an arachidonic acid metabolite and was demonstrated to play a central role in inflammatory and metabolic diseases [34]. It is thus included in the arachidonic acid pathway and together with the linoleic acid pathway (described below) pones the regulation of essential fatty acids metabolism as a plausible key mechanism contributing to the development of the longevity phenotype in the Italian population. To our knowledge, this is the first case representative of a past adaptation evolved in response to specific pathogen-related selective pressures that may have favoured the maintenance in a present-day gene pool of variants secondarily involved in longevity.

To identify further gene networks that may underlie the longevity phenotype in Italians, we also conducted pathway analyses by means of i-GSEA4GWAS. Four KEGG pathways (*i.e.* inositol phosphate metabolism, homologous recombination, linoleic acid metabolism, drug metabolism cytochrome P450) were thus ranked as significantly enriched (FDR ≤ 0.05) in the obtained list of candidate longevity-associated SNPs (for a complete list of significant pathways see Table 4S, all the significant genes in the pathway/gene sets are also reported in Table 5S). Interestingly, a recent paper [35] pointed out similar results when analyzing a different cohort of Italian centenarians, suggesting appreciable genetic influence on the related metabolic profiles. In particular, the authors suggested that Italian centenarians are characterized by a peculiar metabolomic profile that promotes cellular detoxification mechanisms through specific modulation of the arachidonic acid metabolic cascade and through enhanced cytochrome P450 (CYP) enzyme activity. Such an effective mechanism might result in the activation of an anti-oxidative response, as displayed by decreased circulating levels of 9-HODE and 9-oxoODE. These represent reliable markers of lipid peroxidation and oxidative products of linoleic acid, whose altered balance with respect to omega3 is known to be implicated in the side effects of the recently adopted pro-inflammatory diets (more details in Supplementary description - Part II). This is an example of how longevity in the Italian population can be reached by individuals who present genetic backgrounds more apt to face the current environmental and cultural changes. Accordingly, we may speculate that the longevity phenotype and the genes/pathways mainly contributing to it are strictly dependent from the historical period.

### Mechanisms of pleiotropy and trade-offs during lifetime

As recently argued, exploring both trade-off-like and conditional effects may considerably improve the understanding of the genetics of human longevity [7]. To this purpose, we exploited the generated data by dividing the Italian controls in two different subgroups according to their age and by analyzing the related allele frequencies trajectories. This approach, which belongs to the so called "gene frequency methods", represents a powerful tool to identify the role of each variant according to the trends showed during aging. In particular, the change of allele frequency with age may follow linear trends (monotonic) or non-monotonic patterns (usually U-shaped patterns or constant trends until a certain age and then linear ones), in which allele frequency decreases at a given age, but then increases, thus reflecting the establishment of trade-offs in the effect of variants at young and old ages. The choice of controls with a wide range of ages (19-85 yrs) has been performed to explicitly test whether the frequency of an allele varies across age, as recently described [35]. Group 1 (*i.e.* individuals that are less than 50 years old) was considered as representative of the healthy Italian population and of the overall genetic variability observable along the Italian peninsula. Group 2 (*i.e.* individuals that are more than 50 years old) was instead expected to have been subjected to mortality selection, as recently demonstrated [16]. This means that among all individuals belonging to a similar birth cohort, those who live until a given age threshold do not represent a random sample since subjects with disadvantageous characteristics are more likely to die off. Finally, Group 3 (*i.e.* individuals older than 100 years of age) could be considered as characterized by a combination of variants (and experienced environment) favorable for human longevity in the context of the overall Italian genomic background.

**Figure 2:**
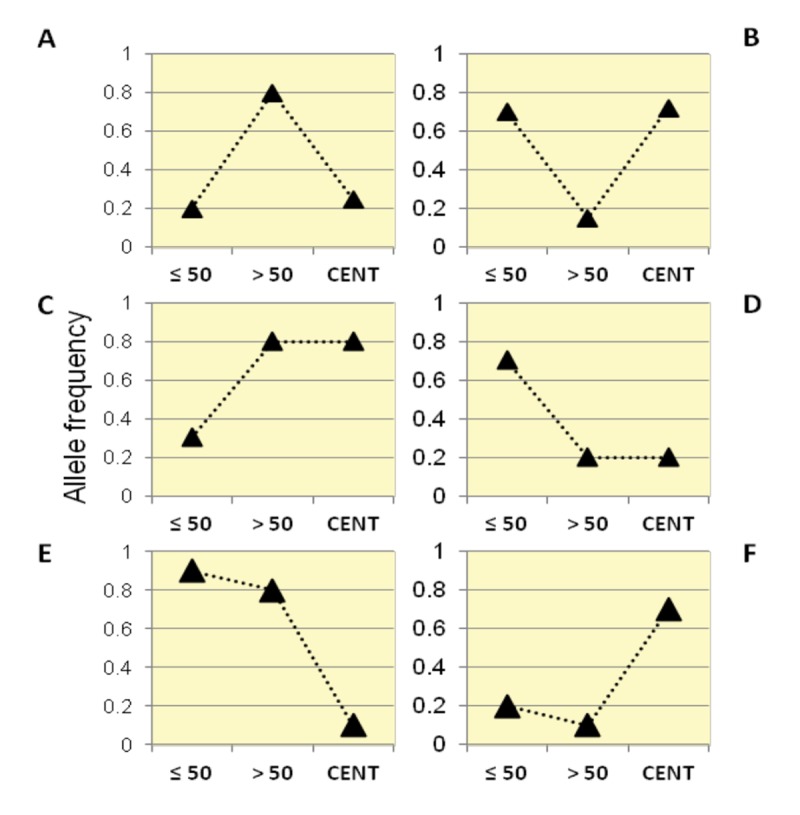
Patterns of allele frequency in the Italian population. SNPs with significant differences in allele frequencies between Group1, Group2 and centenarians (Group 3) were divided into six different categories (Class **A**, **B**, **C**, **D**, **E**, **F**) according to their frequency trajectory over the three examined age intervals.

By following this framework, we identified six categories of variants according to their trajectories of allele frequencies in the three examined groups (Figure 2).

Class A: SNPs for which Group 2 showed higher allele frequencies than Group 1 and centenarians, while similar allele frequencies were observed in centenarians and Group 1. It is thus supposed that such variants are beneficial in Group 2, but not for longevity (also called "from good to bad").

Class B: SNPs for which Group 2 showed lower allele frequencies than Group 1 and centenarians, while similar allele frequencies in centenarians and Group 1 were observed. It is thus supposed that such variants are beneficial for longevity (also called "from bad to good").

Class C: these variants significantly increased in frequency in Group 2 and in centenarians if compared to the general population (Group 1).

Class D: these variants significantly decreased in frequency in Group 2 and in centenarians if compared to the general population (Group 1).

Class E: these variants significantly decreased in frequency in centenarians, while Group 1 and Group 2 showed similar frequencies.

Class F: these variants significantly increased in frequency in centenarians, while Group 1 and Group 2 showed similar frequencies.

According to the proposed classification, we identified 51 SNPs in Class A, 107 in Class B, 274 in Class C, 329 in Class D, 75 in Class E and 67 in Class F.

In Class A (Table 6S), the four most significant SNPs were mapped in the *ESRRG* (estrogen related receptor gamma) locus (rs2576234, rs2032028, rs1436897, and rs2576258, with combined p-values 6.72*10^-7^, 1.36*10^-6^, 2.02*10^-5^, and 4.14*10^-5^, respectively). The rs2576234-C, rs2032028-A, rs1436897-G, and rs2576258-C alleles turned out to be protective against mortality between ages 50 and 85 and then were negatively associated with the probability of becoming centenarian. These candidate SNPs were identified here for the first time and this gene codes for a member of the estrogen receptor-related receptor (ESRR) family. Up-regulation of *ESRRG* was previously linked to cancer, as well as to enhanced neurite outgrowth, learning and memory. These effects indicate the presence of a trade-off between tumor suppression and brain regeneration influenced by *ESRRG*. Such a trade-off could contribute to the Class A trajectory of allele frequencies because it may differentially influence survival at the different age intervals. For instance, cancer (all sites) and neurodegenerative disorders, such as Alzheimer's disease, have their peaks of incidence rate at different ages (before and after the age 85, respectively), which means that these disorders may have their biggest impact on the mortality risk at different ages too. If so, genetic variants with a trade-off-like influence on these disorders could be associated with reduced total mortality risk in middle life, due to cancer, and with increased mortality at the oldest-old ages, due to neurodegenerative disorders (see additional examples of these genetic trade-offs in [7,36,37]. Although it is beyond the scope of this study to investigate the effect of differential fertility of different genotypes over time [34], it is interesting to note that ERR (the homologous of human *ESRRG in Drosophila),* is required to maintain male fertility [35], thus suggesting its potential role in the trade-off reproduction/survival. Some studies showed how some environmental molecules from modern human niche (*e.g.* bisphenol A, nitrated and chlorinated metabolites) strongly bind to *Esrrg receptor* [36]. According to this view, future studies are needed to accurately explore the potential trade-off between reproduction and survival (in the light of energy allocation theory) and its role in shaping life history trajectories in modern environments.

When considering Class B (Table 7S), we identified four highly significant SNPs in the *EPCAM* locus (rs3923559, rs3924917, rs1126497, rs10185866; combined p-values 1.39*10^-6^, 5.34*10^-6^, 7.27*10^-6^, 1.86*10^-5^, respectively). These SNPs show a differential impact on the expression of two genes, *MSH* and *EPCAM* (Table 8S) in different tissues. The former is a protein that recognizes errors in the genome sequence during replication; the latter has instead many functions, such as negative regulation of apoptotic processes and cell-cell adhesion mediated by cadherin, positive regulation of cell motility and cell proliferation, stem cell proliferation and differentiation. The rs3923559-G allele is positively associated to the expression of both *EPCAM* and *MSH* genes and is deleterious (we observed a decrease in allele frequencies) between ages 50 and 85. This is probably due to a trade-off between cell proliferation, apoptosis and repair mechanisms in different tissues and then the same allele becomes protective for longevity (*i.e.* the same allele in a different internal environment and at different ages may have a beneficial effect).

As regards the variants belonging to Class C (Table 9S) and Class D (Table 10S), they seem to have an effect in the middle ages, being instead "neutral" in advanced ages. In Class C, we also identified a SNP (rs17596705) located in *COL25A1* a gene identified in the rare variant analysis conducted by Erikson and colleagues [37], who sequenced the whole genomes of individuals >80 years old with no chronic diseases. In particular, such a variant significantly increased in frequency in Group 2 and in Italian centenarians if compared to the general population.

Moreover, we identified multiple significant SNPs at the *ATP8B4* gene (eight SNPs in Class C: rs6493392, rs11632984, rs11854435, rs2899446, rs11631945, rs8041864, rs7173644, rs6493393 - combined p-values 8.47*10^-7^, 5.23*10^-6^, 6.14*10^-6^, 6.42*10^-6^, 1.52*10^-5^, 7.36*10^-5^, 3.63*10^-4^, 4.00*10^-4^, respectively, and four SNPs in Class D: rs8040030, rs6493402, rs12916154, rs12148767 - combined p-values 3.46*10^-7^, 4.27*10^-5^, 1.50*10^-4^, 5.27*10^-4^). This locus is involved in phospholipid transport in the cell membrane. We observed high frequency of rs6493392-A, rs11632984-G, rs11854435-G, rs2899446-G, rs11631945-A, rs8041864-G, rs7173644-G, rs6493393-A alleles between ages 50 and 85 and in centenarians indicating a beneficial effect of these alleles after reproductive age. The opposite trend was observed for rs8040030-A, rs6493402-G, rs12916154-G, rs12148767-C alleles after reproductive age. Moreover rs8040030-A, rs6493402-G and rs12148767-C are associated to a reduce expression of *ATP8B4* (GTEx) and a significant association between Alzheimer’s disease and the *ATP8B4* locus was reported for rs10519262 [38]. Li and colleagues proposed that rs10519262-GG/GA (associated to higher expression level of *ATP8B4* according to GTEx) is related to increased probability of Alzheimer’s disease onset by age then rs10519262-AA (associated to lower expression of *ATP8B4* according to GTEx). This represents an example of pleiotropy where variants associated to low level of *ATP8B4* expression seem deleterious after the reproductive age, but published data support the fact that in the last decades of life individuals with variants associated to low *ATP8B4* expression reduced the probability of Alzheimer disease onset.

In Class D (Table 10S), we also identified 11 significant SNPs located in the *SORCS2* and *SORCS3* genes, which are members of the vacuolar protein sorting 10 (Vps10) family of receptors that play pleiotropic functions in protein trafficking and intracellular/intercellular signaling in both neuronal and non-neuronal cells [38]. This family of receptors has been implicated in diseases of different aetiology and its variants are considered as genetic risk factors for sporadic and autosomal dominant forms of neurodegenerative diseases, including Alzheimer's disease, frontotemporal lobar degeneration, and Parkinson's disease, as well as for type 2 diabetes mellitus and atherosclerosis. The rs4689642-G, rs4689090-A, rs12646898-G, rs16839840-A, rs4689093-G alleles in *SORCS2* and the rs17118067-A, rs6584635-A, rs7080542-G, rs10884071-A, rs7073438-A, rs1881224-G alleles in *SORCS3* showed a reduced frequency after the age of 50, thus becoming risk alleles in the post reproductive period.

Class E (Table 11S) included significant SNPs in the *CLSTN2* and *ANO3* genes, which have been already described above as candidate loci pointed out by both association and pathway analyses. This category included also rs157582 (combined p-values = 5.99*10^-4^), a SNP located in the *TOMM40* locus and extensively described to be associated to diastolic blood pressure, high density lipoprotein cholesterol measurement, high levels of triglycerides (A allele) and Alzheimer diseases [39–41]. The rs157582-A allele was found to be beneficial (or neutral) until 85 years, while its frequency decreases in centenarians indicating a potential deleterious impact for reaching longevity.

Finally, Class F (Table 12S) included significant variants at genes described above, such as *SMARCA2*, *IRAK2*, and *NACAD*, as well as two SNPs (rs16578 and rs6505393 combined p-values 5.84*10^-6^ and 3.19*10^-4^, respectively) that mapped on the *ASIC2* locus. This gene seems to play a role in neurotransmission and it has been described as a plausible candidate locus in a meta-analysis of four GWASs of survival to age 90 years or older [42] and in a recent paper focused on Chinese centenarians [43].

Overall, the analysis of SNPs allele frequency per age class identified promising candidate loci for further studies. In particular, more data and biological validation on longitudinal cohorts of centenarians are needed to better clarify the role of these genes in shaping complex gene-environment (GxE) interactions.

## CONCLUSIONS

A large body of current biomedical research aims at discovering the genetic determinants of human longevity. However, the strategy applied by traditional GWAS focused on common diseases (i.e. increasing sample sizes) is not applicable in such a peculiar case because long-living individuals are rare in human populations. Accordingly, the vast majority of the GWAS studies performed so far to address this issue put a great deal of effort in performing huge meta-analyses based on considerably different cohorts to reach high statistical power. Unfortunately, this practice completely neglects the population-specific dynamics that may influence the longevity phenotype. We are thus aware that our cohort does not include such a high number of individuals, but we believe that a population-centric perspective is essential to consider also the peculiar gene-environment interactions that are characteristic of each human group.

Interestingly, the definition of longevity has been recently revised by Sebastiani et al. [18] to increase the power to identify statistical significant associations in genetic studies and one percentile survival was the threshold suggested to maximize the probability to detect genetic association with longevity [18]. Here, we considered centenarians (who belong to one percentile survival based upon reference birth cohort for Italy) and a second cohort (HRS) in order to maximize the probability to identify reliable signals, by taking into account that - for the study of longevity - small effect alleles need to be considered and not filtered out. Therefore, we applied an innovative diachronic approach to investigate the genetic determinants that contribute to human longevity showing that this phenotype results from the tangled interaction of demographic processes and adaptive events occurred at the population level with the changing trade-offs experienced by single individuals during different phases of their life.

**Figure 3:**
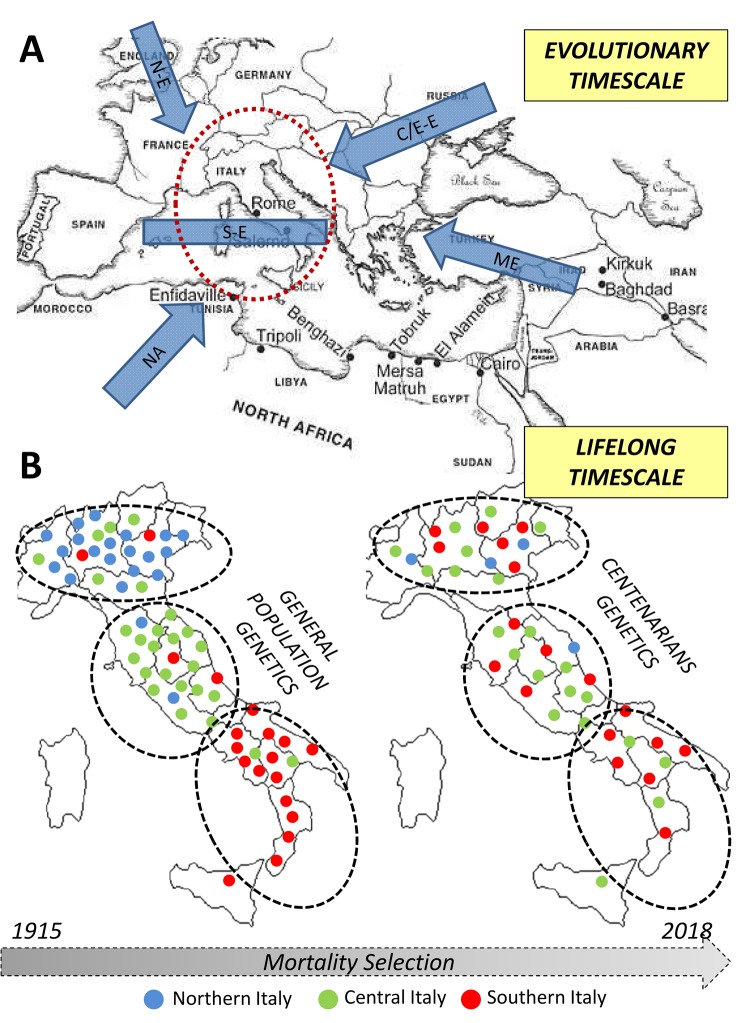
Overview of the diachronic approach used to combine information about processes occurred at different timescales (i.e. evolutionary and lifespan ones) in the study of the genetics of human longevity. (**A**) Northern European, N-E; Central/Eastern European, CE-E; Southern European, S-E; Middle Eastern, ME; Northern African, NA. (**B**) Each dot represents an individual in the general population - left - and in centenarians - right. The colour indicates the recruitment center and the position in the map indicates the genetic similarity).

The genetic bases of longevity are thus highly population-specific and dependent from demographic, biological and environmental dynamics acting over different timescales (*i.e.* during the population evolutionary history and during individuals’ lifespan, as indicated in Figure 3). In fact, we showed that an ancestral component of the Italian genomic background is enriched in the centenarian genomes. Its peculiar distribution in the overall population was plausibly shaped by pre-Neolithic migrations associated to post-LGM human re-expansion from central/southern Italian glacial refugia, being subsequently reduced especially in Northern Italian groups by gene flow associated to Bronze Age demographic processes. However, the fact that centenarians maintained increased proportion of such a genetic component with respect to control subjects and also presented closer genetic affinity to people from Central/Southern Italy than to Northern Italians does not indicate that longevity-associated variants are exclusive of this signature and that longevity is more represented in central/southern regions of the peninsula. More likely, these findings could be interpreted as hints supporting the fact that variants involved in this phenotype, along with those characteristics of the above-mentioned genetic signature, were introduced in the Italian gene pool in considerably ancient times.

The socio-economic structure of the Italian population varies from Northern to Southern Italy and many factors are known to impact on individuals’ mortality (e.g. in Southern Italy the life expectancy is 79.9 years for males and 84.4 years for females, while in Northern Italy it is 81.1 years for males and 85.6 for females). Interestingly, a recent study showed that when controlling for the typology of pension benefit and socioeconomic conditions, people from Northern Italy are characterized by a higher mortality risk compared to populations from Central Italy, while no significant differences between Central and Southern Italians emerged [44]. We can thus speculate that Central and Southern Italians may have got a head start in the race for longevity, but that present-day local socio-economic status hides or alters the impact of longevity-associated genetic components.

Another possibility is that dietary habits may influence the observed patterns. However, this explanation is in our view less plausible because all Italian individuals follow a Mediterranean diet (although with a high level of local diversity from Northern to Southern Italy). Moreover, we identified a SNP associated to longevity (*PTGR1,* rs1053959) that represents an adaptive locus evolved in response to past specific pathogen-related selective pressures and that plays a role in regulation of the arachidonic acid metabolism. Coupled with the candidate loci detected by pathways analyses and belonging to the linoleic acid and cytochrome P450 pathways, such a genetic signature seems to relate to modulation of fatty acids metabolism and to a reduction of the side effects of recently adopted Western pro-inflammatory diets, thus representing an invaluable prerequisite to develop the longevity phenotype in the Italian population. We know that p-values reported in this study are not below the traditional genome-wide significance threshold (i.e. p-value < 10^-8^), but we aimed at highlighting the importance of a high number of small-effect alleles as previously demonstrated for longevity [45]. Finally, by classifying the identified candidate genes according to their potential pleiotropic role during individuals’ life, we demonstrated that becoming centenarian also depends from a dynamic interaction between many genes and the environment (internal and external), which profoundly changes during human lifespan resulting in different trajectories in allele frequencies.

That being so, we can thus speculate that the longevity phenotype could represent an example of "phenotypic convergence" [46] reached by means of both different genetic backgrounds that have been heavily shaped by the evolutionary history of each human population and the changing gene-environment interactions experienced by single individuals during their life, as here described for the Italian population.

## MATERIALS AND METHODS

### Sample collection and genotyping

Centenarians (N = 333, mean age: 100.4 ± 1.4; 258 F, 75 M) were sampled at three different Italian recruitment centres in Northern Italy (N = 66), Central Italy (N = 176) and Southern Italy (N = 91). The present study was designed and performed in accordance with relevant guidelines and regulations and according to ethical principles for medical research involving human subjects stated by the WMA Declaration of Helsinki. DNA samples were recruited after the approval by the Ethical Committee of Sant’Orsola-Malpighi University Hospital (Bologna, Italy). Further approval for this study was also released in January 2011 by the Azienda-Ospedaliera Arcispedale Santa Maria Nuova Ethics Committee (Reggio Emilia) within the framework of the project “GWAS of psoriatic arthritis in the Italian population”. As Italian control group, 773 unrelated healthy individuals representative of the overall Italian population were selected by following strict biodemographic criteria to include only subjects at least three-generations native of a given district, with all grandparents originating from the same province [13,25]: 358 females (F) (mean age: 56.2 ± 11.5 years) and 415 males (M) (mean age: 43.7 ± 10.8 years). Among them, 381 individuals were ≤ 50 years old (F = 101; M = 280) and 392 individuals were > 50 years old (F = 124; M = 268). Information about the sampled Italian provinces, geographic distribution of the control subjects included in this study, and ethical approval for their sampling and genomic characterization are reported in [13]. Genomic DNA was extracted from blood samples using QIAamp 96 DNA Blood Kit. DNA quantification was performed using the Quant-iT dsDNA Broad-Range Assay Kit (Invitrogen Life Technologies, Carlsbad, CA, USA) or by Quant-iT PicoGreen dsDNA Assay Kit (Thermo Fisher Scientific) according to manufacturer protocols. Then 200 ng of DNA were genotyped for 542,585 genetic markers with the Illumina (San Diego, CA, USA) CoreExomeChip v.1.1 array. The HRS is an ongoing panel survey of a nationally representative sample of men and women older than 50 years in the United States. The HRS conducts core interviews every two years using a mixed-mode design of telephone and face-to-face interviews (Recruitment and Retention of Minority Participants in the Health and Retirement Study).

### Statistical analyses

Quality controls (QC) were performed on the generated data to avoid the identification of false positive results when searching for loci potentially involved in longevity and according to the pipelines described in [47] (Supplementary description - Part III). Association analysis was performed by means of the PLINK package v.1.06 using a logistic model and adding sex as a covariate. The QQ and Manhattan plots were calculated using R (package: *qqman*). eQTL analysis was performed by means of the GTEx browser by searching for all eQTL tissues (N ≥ 70) and listing P-Value, Effect Size and Tissue.

To investigate intricate networks of interactions between SNPs and to dig up a biological interpretation of the obtained results we performed pathway analysis. This approach is fundamental in the study of complex traits, such as longevity, where each of the identified candidate SNPs normally has a small phenotypic effect. Pathway analyses were performed by means of i-GSEA4GWAS v.2 [48] by considering all the SNPs in the CoreExome microarray and as recently described in [13]. The used algorithm combined the list of p-values according to SNP-mapped genes and then filtered the collection of pathways/gene sets to obtain a general p-value for each pathway. Only KEGG pathways were finally reported in Table 4S.

For the analysis of patterns of allele frequency in the Italian population an *ad hoc* script was used to divide the examined cohort into three different groups: Group 1 included individuals that are less than 50 years old, Group 2 included individuals that are more than 50 years old, Group 3 included the centenarians. Pairwise associations (Group 1 vs. Group 2; Group 1 vs. Group 3; Group 2 vs. Group 3) were calculated by using a logistic model and adding sex as a covariate. All the SNPs with nominal p-values < 0.01 were divided into six classes (Class A, B, C, D, E, F) according to their allele frequency patterns.

PCA was performed using the “lsqproject = YES” function implemented in the EIGENSOFT package v6.0.1 to project centenarians on the PCA space defined by the Italian control dataset and to overcome potential bias due to the presence of longevity-related variants in the centenarians genomes.

Posterior membership probabilities were calculated for centenarian samples and averaged per sampling location via DAPC (Jombart et al. 2010), using the R *adegenet* package and based on the retained discriminant functions. Such an analysis derived probabilities for each centenarian to belong to different Italian population groups. For this purpose, we considered centenarians as a supplementary group, which does not participate in constructing the model, but which was instead predicted from the model build according to the variation proper of the general population.

Estimates of ancestry proportions were finally obtained using the ADMIXTURE unsupervised clustering algorithm [49]. Probabilistic assignment of each individual to K = 2 through K = 10 hypothetical ancestral populations was calculated and cross validation (CV) procedure was applied to identify the number of clusters for which the model has the best predictive accuracy (K = 5), as supported also by (13). The robustness of the analysis was assessed running fifty replicates with different random seeds for each K tested and consequently monitoring log-likelihood convergence. This analysis included also 50 Mediterranean and European human populations that are reported in Table 13S.

Loci with signatures of local adaptations in the Italian population were finally retrieved from those recently identified [13].

### Editorial Note

The paper was accepted based in part on previous peer-review (by another journal) and the rebuttal, as well as additional expedited peer-review in Aging.

## Supplementary Material

Supplementary Material and References

Supplementary Figure

Supplementary Tables
