# Supplementary material for: Impact of demography and population dynamics on the genetic architecture of human longevity
Source: Aging (Albany NY). 2018 Aug 8;10(8):1947–63. doi: 10.18632/aging.101515 (PMC6128422; doi:10.18632/aging.101515)
Supplement: Supplementary Material and References [file aging-10-101515-s001.pdf]

SUPPLEMENTARY MATERIAL

Part I - Association analyses on Italian centenarians

The association analysis has been performed by logistic regression adding sex as covariate. A total of 29 SNPs were thus identified as nominally significant (p-value < $1*10^{-4}$) and six of them map in candidate genes (i.e. CLSTN2, TBX18, SMARCA2, ANO3, ANKS1B, ASIC2) identified also in the health and retirement study (HRS), adding longitudinal information.

The chromosomal region represented in the list of nominally significant loci is located in the SMARCA2 gene (rs7866316 p-value = $1.32*10^{-5}$) and included also three SNPs in the NACAD gene (rs3735495, rs61740895, rs3735494), with a p-value of $1.52*10^{-5}$, $2.19*10^{-5}$ and $2.38*10^{-5}$, respectively. The SMARCA2 gene was identified as a potential longevity-related locus in a previous meta-analysis of genome-wide association studies including 25,007 participants over 55 years of age (also considering the HRS cohort) and followed-up for over 10 years for mortality and for over eight years for event-free survival. The NACAD gene is instead known to be involved in the inappropriate targeting of non-secretory polypeptides to the endoplasmic reticulum (ER). Then, the other SNPs reported in Table 2S affect functional pathways crucial for longevity and, in particular, rs16578 is located in the ASIC2 gene that plays a role in neurotransmission. The same gene was identified as a plausible candidate longevity-related locus in the paper by Newman and colleagues , who performed a meta-analysis of four genome-wide association studies focused on survival to age 90 years or older. Of the 273 identified SNPs none reached significance after correcting for multiple testing, but ASIC2 was significant at a threshold of 10-4). rs4683806 located in the gene CLSTN2 that was previously associated with cognitive function and linked to synaptic plasticity, memory and connectivity between brain regions . Also the gene ANKS1B (rs10860525) may exert a role in normal brain development, and in the pathogenesis of Alzheimer's disease, in particular interacting with the amyloid beta protein precursor and being expressed in brain and testis. rs4684672 (p-value = $5.28*10^{-5}$) maps in the IRAK2 gene (IL-1R-associated kinase). This gene is involved in the activation of TNF and induction of other citokines and could play a role in immune senescence, being also included in the list of differently expressed genes in early versus replicative senescent and CMV-infected versus untreated human lung fibroblasts [1]. The ANO3 gene linked to endoplasmic reticulum-dependent calcium signalling, a process already linked to longevity [2], showed nominal significant results and its deletion is known to cause increased pain sensitivity in the rat model system. Finally TBX18 is involved in developmental process and it works with other factors, such as Tbx3, form regulatory units that specify the cell types within the hearth. Tbx3 has been identified in a recent paper published by Zeng and colleagues that analyzed sex differences in genetic association with longevity in the Han Chinese population as it is involved in female longevity [3].

eQTL analysis was then performed by considering all the nominal significant SNPs reported in Table 2S. Seven SNPs (rs4684672, rs13215600, rs860844, rs61740895, rs3735494, rs3735495 and rs12985909) were predicted to have an eQTL effect (significant single-tissue eQTL p-value) in different tissues as tested through the GTEx browser and indicating at least a potential pleiotropic effect [4].

We replicated the same (logistic regression) considering only females (N=258) and since female centenarians constitute the majority of the cohort as expected the list of SNPs overlap with the one described above. We did not perform the analysis considering the subgroup of male centenarians because of the small sample size (N=75).

Interestingly, the APOE-e4 and rs4420638 variants, which were identified in many studies on human longevity, did not show significant association with such a trait in the Italian population, as expected also according to the results obtained by recent studies that include populations from Southern Europe [5]. In fact, it is likely that the low frequency of the APOE-e4 allele in Southern Europeans and their peculiar gene-diet interactions (the Mediterranean diet is one example) influence association levels of APOE with longevity in these populations.

Part II - i-GSEA4GWAS analysis

Four KEGG pathways (i.e. inositol phosphate metabolism, homologous recombination, linoleic acid metabolism and drug metabolism cytochrome P450) were thus ranked as significantly enriched (FDR $\leq$ 0.05) in the examined list of candidate longevity-associated SNPs.

Linoleic acid (i.e. fatty acid ω-6) and especially the balance between omega6 and omega3 are known to be implicated in the side effects of the recently adopted pro-inflammatory diets. In fact, it has been estimated that in the last 100-150 years, the absolute and relative changes of ω-6 and ω-3 fatty acids in the food supply of Western societies led their diets to reach a ratio of 20:1 (ω-6/ω-3). On the contrary, such a ratio is supposed to have been of 1:1 during most of human evolutionary

history [6,7] . Based on these data, a number of studies suggested that this modern imbalance in fatty acids ratio may underlie an increased risk of cardiovascular and other chronic diseases, particularly in individuals that are genetically predisposed.

Another identified significant pathway was that related to homologous recombination, which is essential for the double-strand breaks repair of DNA. For instance, it has been demonstrated the link between recombination and carcinogenesis, and some authors proposed homologous recombination functions as a secondary step in tumor progression [8] . Moreover, normal chronic exposure to a variety of environmental and endogenous damages is also supposed to increase the frequency of homologous recombination, thus playing a role in producing high level of heterogeneity in many tumors. Therefore, the obtained results seem to be in with previous considerations about the trade-offs between cancer and aging [9,10].

Part III - Quality controls

Quality controls (QC) were performed on the generated data in attempt to avoid the identification of false positive results when searching for loci potentially involved in longevity and according to protocols and pipelines described in Anderson et al. (2011). Based on genotypes from the X chromosome, for each sample the homozygosity rate was calculated and individuals were classified as males or females (typically males have a X homozygosity rate of 1 and females of less than 0.2). Then identification of individuals with outlying missing genotypes or heterozygosity rate was performed. In particular, individuals with an increased or reduced proportion of heterozygote genotypes were excluded to avoid samples with possible DNA contamination or inbreeding. All individuals with a genotype failure rate > 0.03 and heterozygosity rate exceeding ± 3 standard deviations from the mean were excluded.

A common assumption for association studies is that all the individuals are unrelated otherwise genotypes within families will be over-represented. To control for such a condition, identity by state (IBS) was calculated for each pair of individuals. To this purpose, regions of extended LD were removed from the dataset and SNPs were pruned so that no pairs of markers in window of 50kb presented an r^2 correlation > 0.2. Then, on the basis of genome-wide IBS values, a degree of recent shared ancestry for a pair of individuals (identity by descent, IBD) was estimated and used to identify monozygotic twins (or duplicated samples), first degree relatives, second degree relatives and third degree relatives. One individual for each pairs showing IBD > 0.1875 was removed from the analysis.

Per-marker QC were also performed with the identification of SNPs with excessive missing genotypes (markers with a call rate less than 95% were removed), low minor allele frequency (MAF < 5%) or violating the Hardy-Weinberg equilibrium (HWE).

SUPPLEMENTARY REFERENCES

1. Wolf J, Weinberger B, Grubeck-Loebenstein B. The immunoregulatory effects of CMV-infection in human fibroblasts and the impact on cellular senescence. Immun Ageing A. 2012; 9:1.
2. zZeng Y, Nie C, Min J, Liu X, Li M, Chen H, et al. Novel loci and pathways significantly associated with longevity. Sci Rep. 2016; 6:21243.
3. Zeng, Yi. Sex Differences in Genetic Associations with Longevity. JAMA.
4. Visscher PM, Yang J. A plethora of pleiotropy across complex traits.Nat Genet. 2016 Jun 28;48(7):707-8. doi: 10.1038/ng.3604.5. Beekman M, Blanché H, Perola M, Hervonen A, Bezrukov V, Sikora E, et al. Genome-wide linkage analysis for human longevity: Genetics of Healthy Aging Study. Aging Cell. 2013; 12:184–93.
6. Simopoulos AP. Overview of evolutionary aspects of omega 3 fatty acids in the diet. World Rev Nutr Diet. 1998; 83:1–11.
7. Alvergne A, Jenkinson C, Faurie C, editors. Evolutionary Thinking in Medicine [Internet]. Cham: Springer International Publishing; 2016. Available from: http://link.springer.com/10.1007/978-3-319-29716-3
8. Bishop AJR, Schiestl RH. Homologous Recombination and Its Role in Carcinogenesis. J Biomed Biotechnol. 2002; 2:75–85.
9. Yashin AI, Ukraintseva SV, Akushevich IV, Arbeev KG, Kulminski A, Akushevich L. Trade-off between cancer and aging: What role do other diseases play? Mech Ageing Dev. 2009; 130:98–104.
10. Ukraintseva SV, Arbeev KG, Akushevich I, Kulminski A, Arbeeva L, Culminskaya I, et al. Trade-Offs Between Cancer and Other Diseases: Do They Exist and Influence Longevity? Rejuvenation Res. 2010; 13:387–96.

SUPPLEMENTARY TABLES

Please browse Full Text version to see the data of Supplementary Tables 1-13 related to this manuscript.
