# Supplementary material for: Impact of demography and population dynamics on the genetic architecture of human longevity
Source: Aging (Albany NY). 2018 Aug 8;10(8):1947–63. doi: 10.18632/aging.101515 (PMC6128422; doi:10.18632/aging.101515)
Supplement: Supplementary Figure [file aging-10-101515-s002.pdf]

SUPPLEMENTARY FIGURE

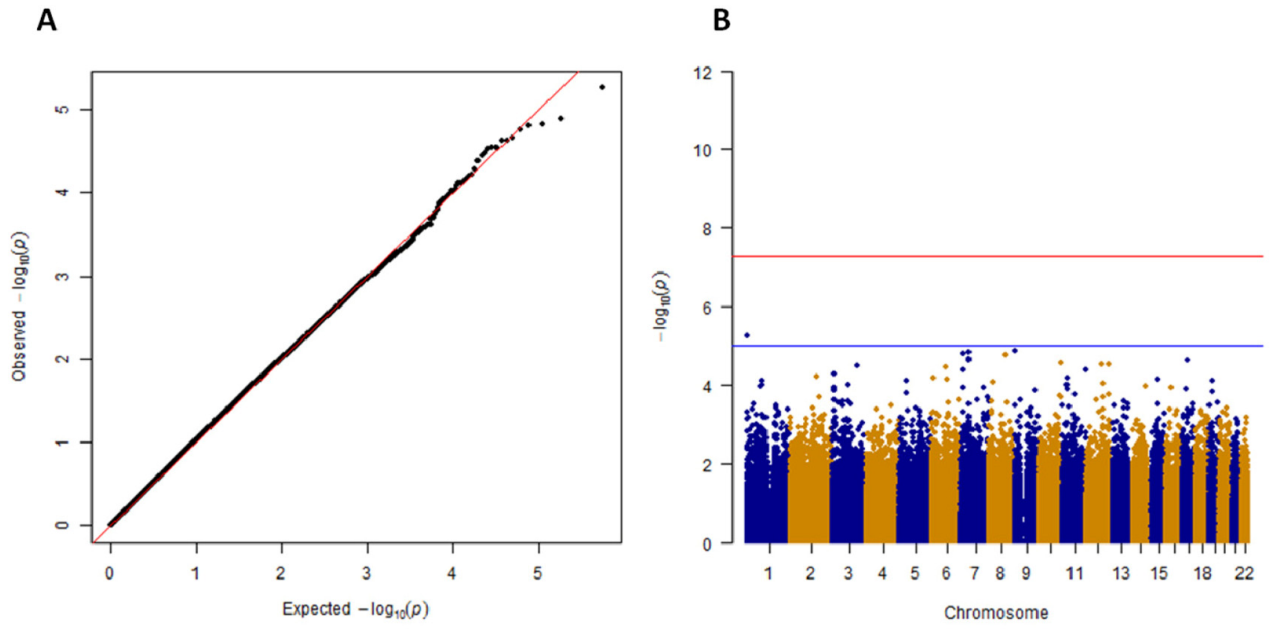

Figure 1S. Association analysis results. (**A**) Manhattan plot of all SNPs tested for the association analysis by considering centenarians and controls. The x-axis shows SNPs according to their chromosomal positions. (**B**) QQplot of expected -$\log_{10}$(p-values) versus observed -$\log_{10}$(p-values). Each variant is reported with a black dot. The genomic inflation factor for this plot is 1.02.
